# Simultaneous Detection of GABA and Glycine Using MEGA‐PRESS With TE Optimization at 3T

**DOI:** 10.1002/mrm.70219

**Published:** 2025-12-08

**Authors:** Justin R. Singer, Kimberly L. Chan

**Affiliations:** ^1^ Department of Biomedical Engineering University of Texas Southwestern Medical Center Dallas Texas USA; ^2^ Advanced Imaging Research Center University of Texas Southwestern Medical Center Dallas Texas USA; ^3^ Department of Radiology University of Texas Southwestern Medical Center Dallas Texas USA

**Keywords:** GABA, glycine, J‐difference editing, MEGA‐PRESS, MR spectroscopy

## Abstract

**Purpose:**

To achieve the simultaneous acquisition of gamma‐aminobutyric acid (GABA) and glycine (Gly) using a MEGA‐PRESS sequence with an optimized TE at 3T.

**Methods:**

MEGA‐PRESS simulations were performed at TEs 60‐88 ms to determine the optimal TE for Gly detection with minimal myo‐Inositol (mI) overlap and maximal GABA detection sensitivity. MEGA‐PRESS data were acquired in the occipital lobe of 6 healthy subjects at TEs of 64 and 68 ms. GABA+ levels, between‐acquisition (SUM (edit‐ON+edit‐OFF) and edit‐OFF) and inter‐subject coefficient‐of‐variation (CVs) and mI, Gly, and glucose CRLBs were evaluated to assess fit reliability. The residuals of the edit‐OFF and SUM fits were compared with and without Gly in the basis set to examine the effect of Gly on fit accuracy and metabolite quantification.

**Results:**

Simulations indicated that optimal Gly detection with minimal overlap from mI is observed at a TE of 64 ms. Simulations and in vivo experiments indicate that this TE resulted in no reduction in GABA+ sensitivity relative to the commonly used TE of 68 ms. Gly between‐acquisition and inter‐subject CVs and CRLBs were substantially lower at a TE of 64 ms than at a TE 68 ms. Spectral fits with Gly excluded from the basis set resulted in a significant increase in CRLBs and fit residuals for mI and glucose at a TE of 64 ms, but not at a TE of 68 ms.

**Conclusion:**

The simultaneous detection of GABA+ from the difference spectrum and Gly from the edit‐OFF/SUM spectra is possible using a MEGA‐PRESS sequence at a TE of 64 ms.

## 1 Introduction

Inhibition in the brain is critical for regulating neural activity. As such, alterations in inhibition have been the subject of numerous studies aimed at characterizing its role in both health and disease and as a potential target for treatment [1, 2]. In the brain, both GABA and Gly function as inhibitory neurotransmitters and can be detected with in vivo ^1^H‐MRS as the concentrations of both metabolites reach millimolar levels. Between the two metabolites, however, GABA has been more widely studied due to its role as the brain's primary inhibitory neurotransmitter, higher concentration in vivo (1–3 mM) [3, 4] and ease of detection. Gly is less commonly measured due to its lower concentration (˜0.7 mM) and high spectral overlap with mI, which complicates quantification. Consequently, the role of GABA in the brain has been better characterized than Gly with in vivo MRS. GABA has been shown to be a major contributor to cognition [5, 6, 7], and altered GABA levels have been detected in many psychiatric pathologies, including schizophrenia [8], autism spectrum disorder [9, 10, 11], and Alzheimer's [12]. At 3T, GABA+ (GABA + macromolecules) is typically detected with J‐difference editing due to the presence of high spectral overlap from larger signals originating from other metabolites such as creatine (Cr).

**FIGURE 1:**
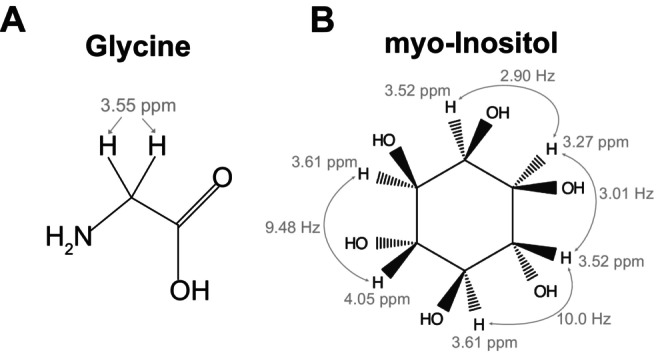
Chemical structures of glycine (A) and myo‐Inositol (B). (A) Glycine has two protons branching from the alpha‐carbon. The two protons are uncoupled and give rise to a singlet peak at 3.55 ppm. (B) Myo‐inositol has 6 non‐exchangeable protons. The relevant chemical shift values are labeled based on proximity to the glycine chemical shift. Scalar (J‐) couplings for these protons are depicted by the arrows.

Although present in the brain at lower concentrations than GABA, Gly plays a significant inhibitory role mainly through its interaction with strychnine‐sensitive glycine receptors [13] and plays a vital role in many neurological processes. Increased Gly concentrations have also been shown to be a biomarker for cancer aggressiveness [14, 15]. Gly has a simple structure with two uncoupled protons branching from the alpha‐carbon which presents in the proton spectrum as a singlet at 3.55 ppm (Figure 1a). Gly cannot be reliably quantified in vivo using conventional MRS scans such as short‐TE PRESS due to the strong overlap from the highly concentrated (4–8 mM) [16] myo‐inositol (mI) signal at 3.5–3.6 ppm [17] (Figure 1b). In gliomas, mI can reach even higher concentrations of ˜10 mM, thus increasing the ratio of mI to Gly to a level higher than that seen in healthy participants [18], and further increasing the difficulty of Gly detection. Despite this substantial overlap, the 3.61 ppm resonance of mI is highly coupled to its 3.52 ppm resonance (˜10 Hz) [19], resulting in significant J‐evolution across TEs. At the same time, the Gly singlet does not evolve due to the absence of J‐coupling. As such, several long TE methods have been developed to take advantage of the J‐evolution of mI with TE to suppress its signal and allow for Gly detection [20, 21].

Due to Gly and GABA's inhibitory actions, it might be desirable to simultaneously detect these metabolites to obtain a comprehensive assessment of the inhibitory processes in the brain. The methods mentioned above, however, require separately tailored scans to robustly detect Gly and GABA at 3T. Many studies require data from multiple regions in the brain and acquiring 5–15‐min scans for each metabolite in each brain region separately can substantially increase the total scan time (e.g., 15–45 min of extra scan time to perform an additional scan in three different brain regions). This limitation thus reduces the amount of information that can be acquired within a single time‐limited scan session of 1–1.5 h.

As such, there has been much interest in optimally detecting multiple molecules in a single edited MRS acquisition using multi‐metabolite editing methods such as HERMES [22, 23, 24] and DEW [25, 26]. Alternatively, the edit‐OFF spectrum from the MEGA‐PRESS scan can be co‐opted to quantify additional metabolites such as myo‐Inositol and *N*‐acetylaspartate [27, 28]. J‐difference editing of GABA is typically performed at a TE of 68 ms; however, GABA has been shown to be measured across a relatively wide range of TEs, ranging from 60 to 80 ms [29] without a loss in sensitivity and thus GABA has often been detected using a slightly longer TE of 80 ms [30] for MM‐suppressed GABA editing. This large range of optimal TEs for GABA detection with MEGA‐PRESS also leaves room for additional opportunities for the simultaneous detection of other metabolites, provided they share the same or similar optimal TEs for detection.

Thus, the goal of this study was to develop a MEGA‐PRESS scan at an optimal TE to robustly measure Gly from the edit‐OFF/SUM spectra and GABA from the DIFF spectrum without the need for additional acquisitions. MEGA‐PRESS simulations were performed at a range of TEs to determine the optimal TE associated with the maximal Gly 3.55 ppm signal, minimal mI signal contamination, and maximal 3.0 ppm GABA+ peak in the DIFF spectrum. This TE was validated in vivo in healthy adults using the MEGA‐PRESS pulse sequence acquired at an optimal TE of 64 ms, as determined from the simulations, and the commonly used TE of 68 ms. The spectra from different TEs were then assessed for differences in Gly fit quality (edit‐OFF/SUM) and GABA+ signal intensity (DIFF).

## 2 Methods

### 2.1 Shared Scan Parameters

All density matrix simulations and in vivo scans were performed at a field strength of 3T using the MEGA‐PRESS [31] pulse sequence consisting of 15‐ms sinc‐Gaussian editing pulses with a bandwidth of 82 Hz. For the MEGA‐PRESS sequence, the 1st editing pulse was placed symmetrically between the excitation pulse and the second refocusing pulse. The 2nd editing pulse was placed halfway between the second refocusing pulse and the start of data acquisition. Other shared sequence parameters included edit‐ON and edit‐OFF frequencies at 1.9 and 7.5 ppm, respectively, a 2000 Hz spectral width, and 2048 points. The minimum reporting standards for in vivo MRS [32] are further described in Table S1.

### 2.2 Simulation Parameters

Density matrix simulations were performed using FID‐A [33] with J‐coupling constants and chemical shifts taken from prior literature [17, 34, 35]. Simulations were performed over a 36 × 36 mm^2^ spatial plane, which was split into 19 × 19 segments. Although the nominal voxel area in these two directions was 30 × 30 mm^2^, simulations were performed beyond this region to capture the transition band of the refocusing pulses that extend beyond the nominal voxel. Simulations were performed using TEs ranging from 60 to 88 ms at increments of 2 ms. All simulations were apodized via a 3 Hz exponential line broadening filter.

### 2.3 In Vivo Parameters

Six healthy volunteers (2 female, 4 male; age 25.8 ± 4 years) gave informed written consent with local Institutional Review Board approval. All scans were performed on Philips Achieva 3T scanner with a 32‐channel receive head coil. T1‐weighted MPRAGE [36] scans were performed for voxel placement. MEGA‐PRESS MRS acquisitions were then acquired at TEs of 64 ms and 68 ms [37]. Prospective frequency correction was performed off the interleaved water reference, which occurred 16 times, once every 22 transients, for each MRS scan as previously described [38]. All scans used VAPOR water suppression [39], a repetition time (TR) of 2 s, first‐order PB (pencil beam) shimming, and a total of 352 transients. Voxels were 30 × 30 × 30 mm^3^ (27 mL) in volume and placed in the occipital lobe.

The in vivo spectra were preprocessed using Gannet 3.1 [40] software using retrospective phase and frequency correction with spectral registration in the time domain [41], HLSVD water filtering as done previously [42], and zero‐filling to 4096 points. The edit‐OFF and SUM spectra were fit using LCModel [43] with a basis set containing aspartate, creatine, cystathionine, GABA, glucose (Glc), glutamine, glutamate, Gly, glycerophosphocholine (GPC), glutathione, mI, lactate, N‐acetylaspartate (NAA), N‐acetylaspartylglutamate (NAAG), phosphocholine (PC), phosphocreatine, phosphoethanolamine, scyllo‐Inositol, taurine, and threonine (Thr). The spectral SNR values were estimated as the maximum Cr peak divided by the standard deviation of the noise from 10 to 12 ppm. Linewidths were estimated as the full width at half max of the total NAA (NAA + NAAG) peak at 2.0 ppm. The difference spectra were fit with Gannet 3.1 [40].

### 2.4 Simulation Analysis

The root mean square (RMS) of the contaminating signals (mI, Glc, Thr), scaled to previously reported in vivo concentrations [17], from 3.5 to 3.6 ppm across a range of TEs from 60 to 88 ms was calculated to find the optimal TE for detecting Gly with minimum contribution from nuisance signals. To evaluate the effect of varying mI levels and linewidths in detecting Gly, noise was added to the simulated OFF and SUM spectra to match the mean SNR of the in vivo spectra (Table 1), Gly was scaled from 0 to 3 mM, and the other metabolites were scaled according to their average in vivo concentration estimated from the edit‐OFF of TE 64 ms. Additional line broadening was applied to reach linewidths of 5 or 10 Hz, which are considered excellent and acceptable linewidths for the brain at 3T [44]. To evaluate the effect of TE on the 3.0 ppm edited GABA peak intensity, GABA‐edited spectra were simulated at TEs ranging from 60 to 80 ms and apodized with a 6 Hz exponential filter to mimic in vivo conditions [44]. GABA‐edited MRS data were simulated with and without an in vivo T2 relaxation constant of 88 ms to evaluate the effect of T2 relaxation on TE‐modulated 3.0 ppm GABA‐edited peak integral [29]. The simulations without T2 relaxation serve as a reference to show how much the TE‐modulated curve shifts when T2 relaxation is accounted for.

**TABLE 1:** SNR and FWHM values for the in vivo spectra.

	TE 64 ms		TE 68 ms	
	Edit‐OFF	SUM	Edit‐OFF	SUM
SNR	247 ± 28	377 ± 64	248 ± 44	365 ± 44
FWHM (Hz)	4.73 ± 0.57	4.85 ± 0.59	4.6 ± 0.49	4.85 ± 1.0

*Note*: The SNR were calculated as the maximum amplitude from the creatine peak divided by the standard deviation of the noise from 10 to 12 ppm, and FHWM was from the *N*‐acetylaspartate peak. Data are shown as mean ± standard deviation.

### 2.5 In Vivo Analysis

For the difference spectra, the areas of the 3.0 ppm GABA+ peak were evaluated to judge the sensitivity in measuring from this peak, including T2 relaxation effects as opposed to the concentrations, which correct for these effects. The GABA+ areas were also evaluated to allow for comparisons to the simulated results. Differences in the fitted Gly concentrations between the OFF and SUM at their corresponding TE were calculated as the absolute difference between the two acquisitions divided by the mean for each subject to assess fit consistency. Cramér–Rao lower bounds (CRLBs) taken from LCModel fits to the data were evaluated to assess fit quality. To further evaluate fit quality and the effect of Gly on the quantification of mI and Glc, the SUM and OFF spectra were fit with two separate basis sets, which were identical except for the inclusion of Gly (Gly+) and the exclusion of Gly (Gly–). Differences in the resulting fit residuals from 3.4 to 3.7 ppm where Gly is detected and mI and Glc CRLBs between fit types at each TE were then assessed. Inter‐subject Gly CVs were calculated as the standard deviation of the fitted Gly levels divided by the mean across all subjects at each TE.

### 2.6 Statistical Analysis

All statistical analyses were conducted in MATLAB. Mann–Whitney *U* tests were used to assess statistically significant differences in CRLBs, CVs, GABA+ areas and concentrations, and fit residuals between TEs.

## 3 Results

**FIGURE 2:**
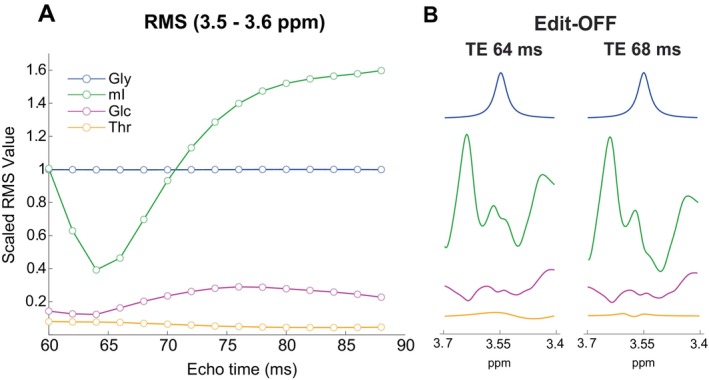
(A) Root mean square (RMS) of MEGA‐PRESS edit‐OFF simulations for Gly, mI, Glc, and Thr signal from TE 60 ms—88 ms. Metabolites were scaled to in vivo concentrations and normalized to the glycine RMS. (B) Gly, mI, Glc, and Thr individual edit‐OFF spectral profiles at 3.4–3.7 ppm range at TE 64 ms and TE 68 ms. For all metabolites, 5 Hz line broadening was applied to mimic in vivo conditions.

The RMS of Gly, mI, Glc, and Thr from 3.5 to 3.6 ppm, scaled to previously reported in vivo concentrations [17, 18, 45], and normalized to Gly as a function of TE, are depicted in Figure 2a. Among the three nuisance metabolites, mI has the highest RMS relative to Gly across all TEs. Across the TEs measured, the RMS of mI reaches a minimum at 64 ms. Notably, the RMS of mI increases by roughly 1.8‐fold from TE 64 to 68 ms. Relative to mI, the RMS of Glc and Thr have substantially smaller fluctuations, with minima at TE 64 ms and TE 84 ms, respectively. These differences can be seen qualitatively in Figure 2b, where the mI spectrum from 3.5 to 3.6 ppm, the region where Gly is detected, is substantially larger at TE 68 ms than at TE 64 ms. In contrast, the Glc and Thr signals remain minimal at both TEs.

**FIGURE 3:**
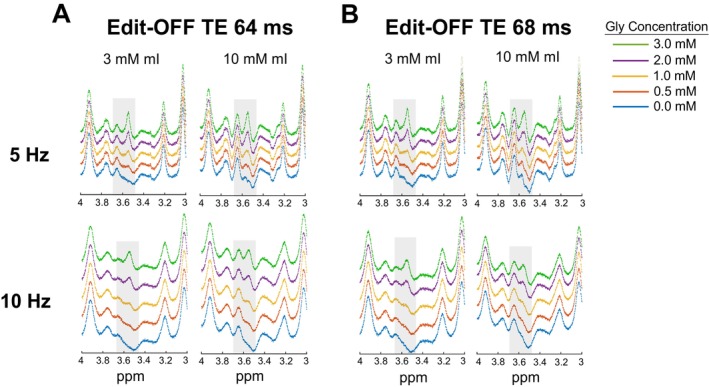
Simulated MEGA‐PRESS edit OFF spectra as a function of TE, line broadening, and Gly/mI concentration ratios for (A) TE 64 ms and (B) TE 68 ms. All metabolites were scaled to in vivo concentrations except for Gly which were scaled to values of 0, 0.5, 1, 2, or 3 mM, and mI, which were scaled to values of 3 (left column) or 10 mM (right column). Each simulation were line‐broadened to either 5 Hz (top row) or a 10 Hz (bottom row). All spectra are shown from 3 to 4 ppm. The highlighted regions are to mark notable differences in Gly and mI signal.

Simulated edit‐OFF and SUM spectra with 5 Hz and 10 Hz linewidths are shown in Figure 3 and Figure S1, respectively. Gly and mI were scaled from 0 to 3 mM and 3 to 10 mM [17, 18], respectively, while the other metabolite levels were scaled according to the average concentrations from the edit‐OFF spectra. At a TE of 64 ms, an increasing Gly peak can be seen at 3.55 ppm as the concentration is increased across both mI concentrations and linewidths. A similar increase in the Gly peak can also be seen at a TE of 68 ms when both the mI concentration and linewidth are low at 3 mM and 5 Hz, respectively. At the same mI concentration, a similar increase cannot be observed when the linewidth is increased to 10 Hz, until the concentration reaches ≥ 1 mM. When the mI concentration is high at 10 mM, visible increases in the Gly peak at a TE of 68 ms cannot be seen until the concentration reaches 1–2 mM for both spectral linewidths.

**FIGURE 4:**
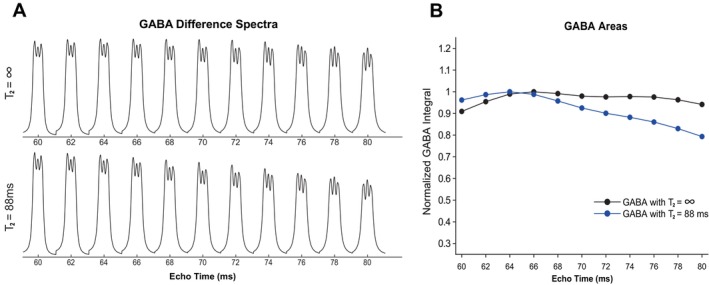
MEGA‐PRESS simulations of GABA performed at a range of TEs. The spectra and area were extracted from 2.79 to 3.28 ppm. (A) GABA‐edited multiplets as a function of TE with and without T2 relaxation effects. (B) The area of the shown GABA‐edited spectra as a function of TE with (blue) and without (black) T2 relaxation effects. Areas were normalized to the maximum of the respective non‐corrected and T2–corrected values.

The simulated GABA‐edited resonance at 3.0 ppm across TEs ranging from 60 to 80 ms, with (top) and without (bottom) taking in vivo T2 relaxation into consideration, is shown in Figure 4a. Consistent with prior work [29], when T2 relaxation is not taken into consideration, the height of the peaks stays consistent across the range of TEs. When T2 relaxation is taken into consideration, the GABA peak height starts to decrease after a TE of 64 ms. This can be seen quantitatively in Figure 4b, where the relative areas display small changes across the range of TEs when T2 relaxation is not taken into account. When T2 relaxation is taken into account, however, the normalized GABA integral starts to decrease at a TE of 66 ms. The GABA area reached a maximum at 64 and 66 ms, with and without T2 relaxation taken into consideration, respectively. This maximum GABA signal at a TE of 64 ms coincides with the TE for minimal overlap of Gly with mI and Glc as seen previously in Figure 2a.

**FIGURE 5:**
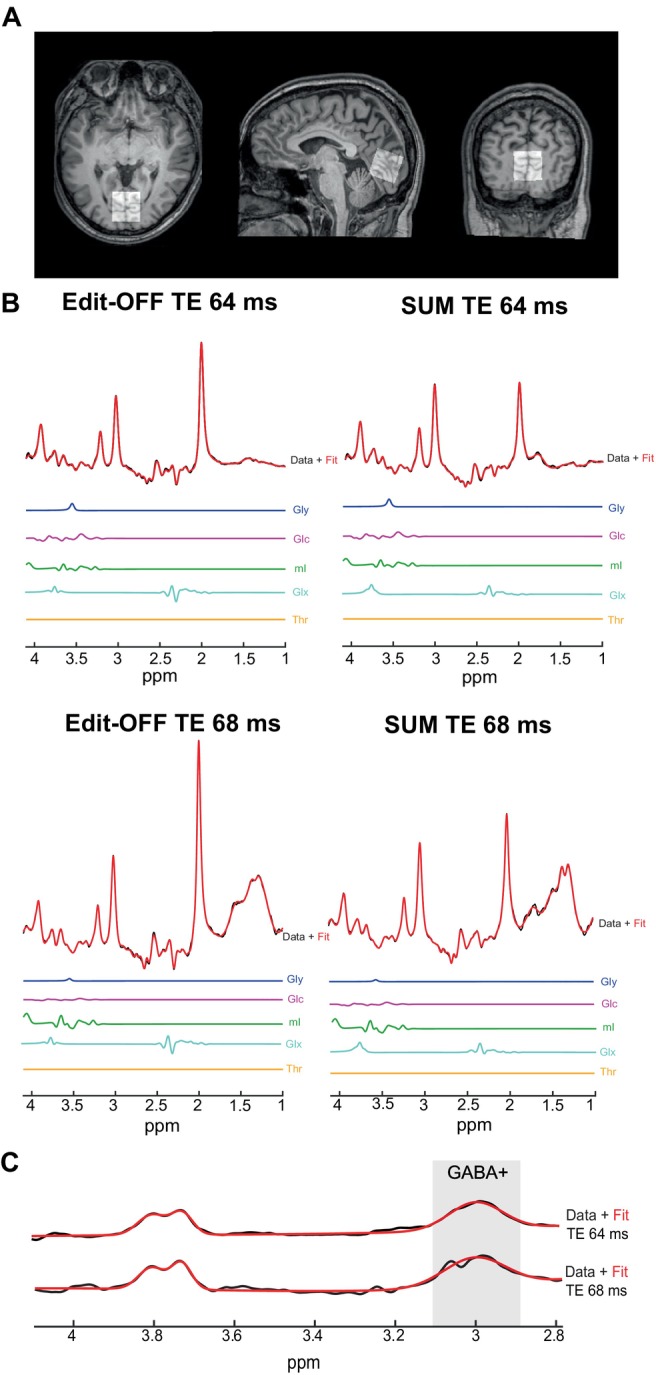
Example in vivo voxel placement, spectra, and fits. (A) Representative MP‐RAGE scan with the corresponding 27 mL voxel placement in the occipital lobe. (B) Example in vivo spectra and fits for Glc, Gly, mI, Glx, and Thr. Spectra are the edit‐OFF and SUM acquired from the respective TE 64 and 68 ms MEGA‐PRESS scans. (C) Representative difference spectra and fits for TE 64 and 68 ms.

Figure 5a shows a representative voxel placement in the occipital lobe. Figure 5b shows representative spectra and their corresponding fits to the edit‐OFF and SUM spectra at both TE 64 ms and 68 ms. Here, it can be seen that the acquired spectra and fits were of high quality, with high SNR and narrow linewidths of ˜5 Hz (Table 1). The SNR in the SUM spectra was ˜50% higher than that in the edit‐OFF spectra at both TEs. These differences are due to the SUM spectra containing twice the number of signal averages as the edit‐OFF and are consistent with the theoretical √2‐fold increase in SNR. At both TEs, the total NAA peak at 2.0 ppm was smaller in the SUM spectra than in the edit‐OFF spectra due to the edit‐ON pulse at 1.9 ppm in the edit‐ON acquisition, which saturates the total NAA peak. Figure 5c shows the corresponding GABA‐edited spectra and fits, which were of high quality with low fit errors of 5.3% ± 1.1% and 6.9% ± 3.1% for TE 64 ms and TE 68 ms, respectively.

**FIGURE 6:**
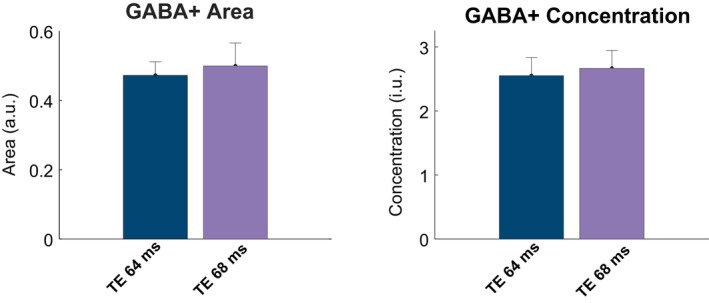
Bar graphs of in vivo GABA+ areas and concentrations for TE 64 (blue) and TE 68 ms (purple) taken from the difference spectra.

**FIGURE 7:**
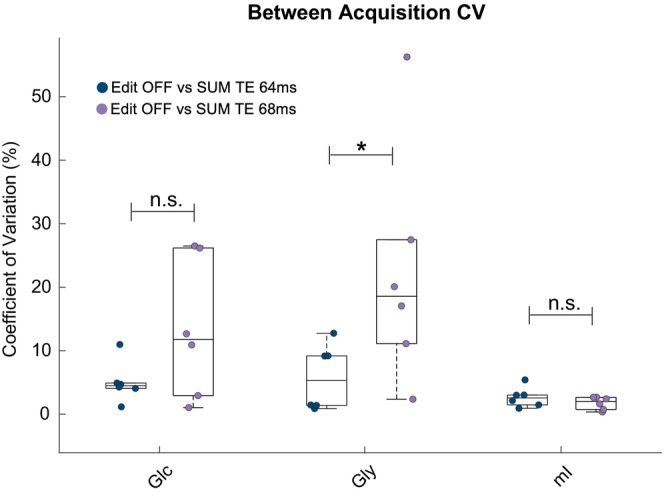
In vivo between‐acquisition coefficient of variation for Glc, Gly, and mI. The variance is evaluated based on the difference between the metabolite concentrations derived from edit‐OFF and SUM spectra for the equivalent echo time (*p < 0.05).

The in vivo area and concentration values taken from the 3.0 ppm GABA+ peak are depicted in Figure 6. The peak areas and concentrations were the same for both TEs, with no statistically significant differences (*p* = 0.699 and *p* = 0.394, respectively). Estimated concentrations and CRLBs from the edit‐OFF and SUM spectra are shown in Table 2. For the unedited spectra, the between‐subject CV of Gly for the edit‐OFF and SUM at TE 64 ms were 19.9% and 6.63%, respectively, which were consistent with previously reported values, which ranged from 8% to 20% [20, 21, 46]. These values were also considerably lower than those at TE 68 ms, which were 37.8% and 44.2% for the edit‐OFF and SUM, respectively. Similarly, the Gly CRLBs were lower in TE 64 ms at 5.2% ± 1.5% and 6.2% ± 1.6% for the edit‐OFF and SUM, respectively, compared to those at TE 68 ms, which were 9.0% ± 9.7% and 14.3% ± 7.6%, respectively, conveying a higher fit quality at TE 64 ms. These differences were statistically significant for the SUM spectra at TE 64 ms vs. TE 68 ms (*p* = 0.011). The between‐acquisition metabolite CVs are shown in Figure 7. Notably, Gly between‐acquisition CV values are significantly lower in the TE 64 ms spectra when compared to the TE 68 ms spectra (*p* = 0.026), whereas the between‐acquisition CVs for Glc and mI display no significant differences between TEs.

**TABLE 2:** In vivo metabolite concentration and CRLB values for edit OFF and SUM spectra.

	TE 64 ms				TE 68 ms			
	Edit‐OFF		SUM		Edit‐OFF		SUM	
	Concentration (i.u)	CRLB (%)	Concentration (i.u.)	CRLB (%)	Concentration (i.u.)	CRLB (%)	Concentration (i.u.)	CRLB (%)
Glc	1.73 ± 0.27	4.67 ± 1.03	1.71 ± 0.25	5.67 ± 0.82	1.81 ± 0.85	8.67 ± 10.46	1.53 ± 0.56	7.83 ± 3.31
Gly	0.88 ± 0.18	5.17 ± 1.47	0.90 ± 0.06	6.17 ± 1.60	0.82 ± 0.31	9.00 ± 7.92	0.52 ± 0.23	14.3 ± 7.66
mI	3.34 ± 0.74	4.17 ± 0.84	3.48 ± 0.73	4.50 ± 0.84	4.70 ± 1.49	4.00 ± 1.27	4.61 ± 1.37	4.16 ± 0.75

*Note*: Data are shown as mean ± standard deviation.

Abbreviations: Glc, glucose; Gly, glycine; mI, myo‐Inositol.

**FIGURE 8:**
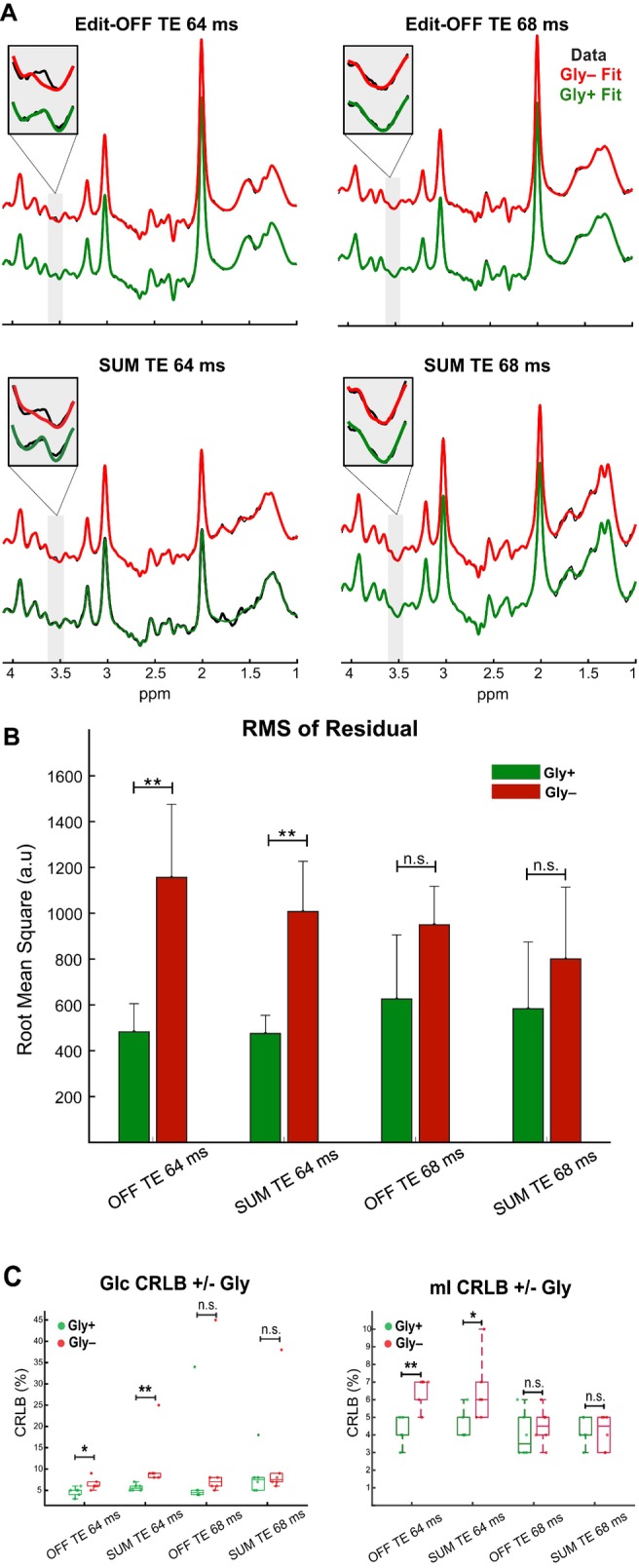
Glycine influence on nearby metabolites. (A) In vivo spectral fits with (green, Gly+) and without (red, Gly−) Gly in the basis set. Each subplot displays a zoomed‐in window to enhance the visibility of the data and fit in the Gly ppm range. (B) Bar graph of the RMS of the residuals from 3.4 to 3.7 ppm fit with and without Gly in the basis set for each spectrum. (C) Boxplots with differences in Glc and mI CRLBs between Gly+ and Gly− fits for each scan type (*p < 0.05, **p < 0.01).

Example in vivo spectral fits with (Gly+) and without (Gly−) Gly in the basis set are shown in Figure 8a. At the highlighted region at ˜3.55 ppm, where a Gly peak is expected, the TE 64 ms Gly– spectra exhibit a notable gap between the data and the fit, which is not present with the corresponding Gly+ fit (Figure 8a). In contrast, no observable difference between the data and fit within the highlighted region was observed between Gly+ and Gly− fits at a TE of 68 ms for both the edit‐OFF and SUM scans, indicating a less reliable Gly fit compared to TE 64 ms. This can be seen quantitatively in Figure 8b, where the fit residual RMS for Gly− was roughly 2.3‐fold higher than Gly+ for edit‐OFF and SUM spectra at TE 64 ms (*p* = 0.002, and *p* = 0.002). In contrast, no difference in fit residual RMS between Gly+ and Gly− was observed for the edit‐OFF or SUM spectra at TE 68 ms (*p* = 0.093, 0.240), indicating a more accurate Gly fit at TE 64 ms. Figure 8c shows the change in CRLBs for Glc and mI when fitted with and without Gly. The CRLBs for Glc and mI are significantly increased by roughly 50% in the Gly− fits for both the edit‐OFF and SUM spectra at TE 64 ms, but not in the Gly− fits for either the edit‐OFF and SUM spectra at TE 68 ms. As such, the exclusion of Gly from the basis set had a significant impact on the Glc and mI fits at TE 64 ms, but not at TE 68 ms.

## 4 Discussion

In this study, it is demonstrated that MEGA‐PRESS data acquired at a TE of 64 ms allows for the simultaneous detection of Gly and GABA from the edit‐OFF/SUM spectra and difference spectra, respectively. Our results may suggest that a TE of 64 ms displays greater Gly fit consistency and quality when compared to a TE of 68 ms without a reduction in GABA+ detection sensitivity. Both GABA and Gly function as inhibitory neurotransmitters, thus making the detection of both metabolites desirable. It has been shown that GABA and Gly are co‐released from synaptic vesicles [47], and Gly and GABA_A_ receptors are colocalized at synapses in spinal cord neurons, where the two regulate one another by crosstalk inhibition [48]. Gly neurotransmission in the forebrain has not been fully characterized but has been suggested to regulate neurotransmission largely through tonic inhibition. Preclinical studies have demonstrated that there is significant Gly inhibitory action via tonic inhibition within the forebrain [49]. Similar work has shown that an induced buildup of Gly leads to a significant increase in strychnine‐sensitive current in the medial prefrontal cortex, causing a decrease in overall excitability of neurons, thus implying a potential dependence on Gly concentration [50]. Notably, the results suggest that GABA and Gly have interlinked roles in inhibition, where GABA is phasic inhibition and Gly is tonic inhibition [50]. Further preclinical studies are needed, however, to aid in the interpretation of the MRS Gly signal and the role that Gly plays in neurotransmission in the brain.

In principle, this method can be used to acquire a more comprehensive panel of inhibitory tone measurements with a singular scan. It should also be noted that the edit‐OFF pulse at 7.5 ppm editing pulse does not affect any upfield metabolites, allowing for the quantification of non‐edited metabolites in the edit‐OFF spectrum. Additionally, the edit‐ON pulse at 1.9 ppm does not affect any metabolite spins near the 3.55 ppm Gly range, allowing detection of Gly from the SUM spectra as well, which is preferable relative to that of the edit‐OFF spectra, considering the ˜42% SNR gain from the greater number of transients.

J‐difference editing with MEGA‐PRESS is considered the gold standard for GABA detection [51]. Gly, on the other hand, has been detected with a range of different techniques, and most of these have typically been performed with long TEs (150–200 ms) to take advantage of the J‐evolution of mI to reduce its signal. Given GABA's short T2 relaxation of 88 ms at 3T [29], these methods would result in a substantial loss of ˜250% GABA+ signal compared to TE 64 ms. In contrast, the proposed method allows for the simultaneous detection of GABA+ and Gly at no cost to GABA+ spectral quality, thus obviating the need to acquire two separate scans for each metabolite and consequently allowing for shorter scan times. Furthermore, the simultaneous detection of these inhibitory metabolites would be highly valuable for functional MRS [52, 53] studies by allowing us to directly assess potential temporal dissociations between metabolite changes and reducing the inconsistencies from stimulus adaptation that would occur if we were acquiring the spectrum for each metabolite separately [54, 55]. To date, functional MRS studies have been more commonly performed to measure GABA [56, 57], and less so for Gly. It has been demonstrated, however, that Gly concentrations in the visual cortex significantly decreased upon visual stimulation at 7 T [58]. Although demonstrated here in combination with J‐difference editing, the optimized TE of 64 ms found here could also be used in conjunction with a non‐edited PRESS scan for Gly‐only detection; such a scan would also grant a theoretical SNR boost of ˜140% relative to the aforementioned long TE scans, assuming a Gly T2 relaxation time of ˜125 ms at 3T [59]. As an added benefit, this method does not require additional hardware or sequences and can be used on any scanner with a pre‐existing MEGA‐PRESS or PRESS sequence simply by adjusting the TE.

To date, a TE of 68 ms continues to be the most commonly used TE to detect GABA+ with MEGA‐PRESS [37]; however, GABA has been measured with high sensitivity across a wide range of TEs [30]. In agreement with this work, no significant differences in signal intensity were found between the simulated GABA peak at TE 64 ms versus that at TE 68 ms, even when taking in vivo T2 relaxation into consideration. It should be noted that the simulations performed did not take the MM signal into account; however, this MM signal difference is expected to be negligible given the 4 ms difference in TE, despite its short T2 relaxation time. This was also observed in vivo, where the GABA+ areas and concentrations were the same between TE 64 ms and TE 68 ms. MMs were also not included in the basis sets used to fit the edit‐OFF or SUM spectra, as there were no experimental MM spectra for TE = 64 and 68 ms acquired in this study. The inclusion of an experimentally derived MM basis, however, could improve the fitting accuracy. Considering this and the data from the present study, which points to potentially improved Gly detection with TE 64 ms versus TE 68 ms, simultaneous detection Gly and GABA+ with MEGA‐PRESS appears to be optimal when performed at a TE of 64 ms.

It should be noted that the FIDs for all scans were zero‐filled prior to fitting, which has been shown to improve model accuracy [60]. This process, however, removes the independence between points and likely affects the resulting CRLB values when fitting with LCM methods. In this study, the CRLB values were not lower in the SUM spectra than in the edit‐OFF spectra despite the higher SNR of the SUM spectra. This is likely due to the sufficiently high SNR of both the edit‐OFF and SUM spectra, as it has been shown that the CRLB estimates of the standard deviations on the measured parameters remain accurate if they are measured beyond a breakdown threshold [61]. It should also be noted that at 3T, it is generally not possible to separate PC and GPC at 3T. Thus, variations in the PC/GPC ratios could affect mI quantification and potentially influence Gly quantification as well. In this study, in vivo validation was performed in healthy controls, which produced spectra of very high quality. Further work is needed, however, to validate the robustness of Gly quantification in clinical populations who are more prone to motion during the acquisition and thus produce data of lower quality.

Despite the small 4 ms difference in TEs, simulations showed a substantially lower overlap of 39% between Gly and mI at TE 64 ms versus 70% at TE 68 ms due to the large J‐coupling (˜10 Hz) between the 3.52 ppm and the 3.61 ppm resonance of mI, the latter of which overlaps with the 3.55 ppm Gly resonance. This difference in spectral overlap resulted in significantly improved Gly measures at TE 64 ms versus TE 68 ms with lower between‐acquisition CVs for Gly at TE 64 ms versus TE 68 ms, and inter‐subject CVs that were more consistent with literature values at TE 64 ms versus TE 68 ms [20, 21, 46]. It should be noted that the TE optimization was performed here with a focus on minimizing the signal overlap between mI and Gly due to the former's high concentration and signal intensity. Besides mI, Glc and Thr also have resonances near the Gly resonance at 3.55 ppm. Both metabolites, however, have low concentrations of ˜1.16 mM for Glc [62] and ˜0.33 mM for Thr [63], which are substantially lower than that of 4–8 mM for mI [16] and thus have minimal effect on the observable Gly signal. Additionally, the CRLBs for Thr as estimated from the in vivo data were very high (45%–999%) and were thus excluded from data analysis; this low fit quality is likely due to its very low in vivo concentration and low signal intensity with only one observable proton between 3 and 4 ppm. In addition, when Gly was excluded from the basis set, Glc and mI CRLBs and fit residuals were found to increase at TE 64 ms but not at TE 68 ms, which may indicate a more robust Gly fit at a TE of 64 ms versus a TE 68 ms.

## 5 Conclusion

In summary, the TE of MEGA‐PRESS was optimized to allow for the simultaneous quantification of Gly and GABA+ in a single scan. A TE of 64 ms was found to be optimal with higher quality Gly fits relative to that at a TE of 68 ms, without a loss in sensitivity for detecting GABA+. The proposed method is easy to implement on any scanner with a MEGA‐PRESS sequence and opens the door for acquiring a comprehensive profile of inhibitory tone within a single scan session and measuring temporal associations/dissociations between metabolites in functional MRS studies.

## Funding

This work was supported by National Cancer Institute (P30CA142543); American Cancer Society (IRG‐24‐1322075‐19‐IRG).

## Supporting information


**Table S1:** Minimum Reporting Standards in Magnetic Resonance Spectroscopy checklist.
**Figure S1:** Simulated MEGA‐PRESS SUM spectra as a function of TE, line broadening, and Gly/mI concentration ratios for (A) TE 64 ms and (B) TE 68 ms. All metabolites were scaled to in vivo concentrations except for Gly which were scaled to values of 0, 0.5, 1, 2, or 3 mM, and mI, which were scaled to values of 3 (left column) or 10 mM (right column). Each simulation was line‐broadened to either 5 Hz (top row) or a 10 Hz (bottom row). All spectra are shown from 3 to 4 ppm. The highlighted regions are to mark notable differences in Gly and mI signal.

## Data Availability

The MATLAB code and data that supports the findings of this study is openly available at https://github.com/justinsinger2/Gly‐GABA‐TE‐OPTIMIZATION.
