# Supplementary material for: Simultaneous Detection of GABA and Glycine Using MEGA‐PRESS With TE Optimization at 3T
Source: Magn Reson Med. 2025 Dec 8;95(5):2479–88. doi: 10.1002/mrm.70219 (PMC12962194; doi:10.1002/mrm.70219)
Supplement: Supplementary file 1 — Table S1: Minimum Reporting Standards in Magnetic Resonance Spectroscopy checklist. Figure S1: Simulated MEGA‐PRESS SUM spectra as a function of TE, line broadening, and Gly/mI concentration ratios for (A) TE 64 ms and (B) TE 68 ms. All metabolites were scaled to in vivo concentrations except for Gly which were scaled to values of 0, 0.5, 1, 2, or 3 mM, and mI, which were scaled to values of 3 (left column) or 10 mM (right column). Each simulation was line‐broadened to either 5 Hz (top row) or a 10 Hz (bottom row). All spectra are shown from 3 to 4 ppm. The highlighted regions are to mark notable differences in Gly and mI signal. [file MRM-95-2479-s001.zip › mrm70219-sup-0001-TableS1.docx]

**Supporting Information Table S1** Minimum Reporting Standards in Magnetic Resonance Spectroscopy checklist

Hardware

| Field Strength | 3T |
| --- | --- |
| Manufacturer | Philips |
| Model (Software) | Achieva, software release 5.7.1 |
| RF Coil | 32-channel ^1^H head coil |
| Additional Hardware | N/A |

Acquisition

| Pulse Sequence | MEGA-PRESS |
| --- | --- |
| Volume of Interest (VOI) | Occipital Lobe |
| Nominal VOI size | 27 mL |
| RF Coil | 32-channel head coil |
| Repetition Time (TR) and Echo Time (TE) | TR = 2 s and TE = 64 and 68 ms |
| Number of averaged spectra | 352 averages |
| Additional Parameters | 2000 Hz spectral width, 2048 spectral points |
| Water Suppression Method | VAPOR |
| Shimming Method | Pencil beam (PB)-auto, 1st order |
| Triggering or Motion Correction | N/A |

Analysis and Data Quality

| Analysis Software | Gannet 3.1, LCModel |
| --- | --- |
| Processing steps deviating from reference | N/A |
| Output Measure | Institutional Units |
| Quantification Reference | Unsuppressed water |
| Reported Variables | SNR (based off the fitted Cr peak), full-width-at-half-max of the fitted NAA peak. |
| Data exclusion criteria | Visual inspection (lack of interpretable spectrum, movement) |
| Sample spectrum | Figure 5b, Figure 5c |

Figure S1) Simulated MEGA-PRESS SUM spectra as a function of TE, linebroadening, and Gly / mI concentration ratios for (A) TE 64 ms and (B) TE 68 ms. All metabolites were scaled to in vivo concentrations except for Gly which were scaled to values of 0, 0.5, 1, 2, or 3 mM, and mI, which were scaled to values of 3 (left column) or 10 mM (right column). Each simulation was line-broadened to either 5 Hz (top row) or a 10 Hz (bottom row). All spectra are shown from 3 – 4 ppm. The highlighted regions are to mark notable differences in Gly and mI signal.
