# Supplementary figures and images for: Simultaneous Detection of GABA and Glycine Using MEGA‐PRESS With TE Optimization at 3T
Source: Magn Reson Med. 2025 Dec 8;95(5):2479–88. doi: 10.1002/mrm.70219 (PMC12962194; doi:10.1002/mrm.70219)

# A SUM TE 64 ms

# B SUM TE 68 ms

Gly Concentration

- 3.0 mM
- 2.0 mM
- 1.0 mM
- 0.5 mM
- 0.0 mM

5 Hz

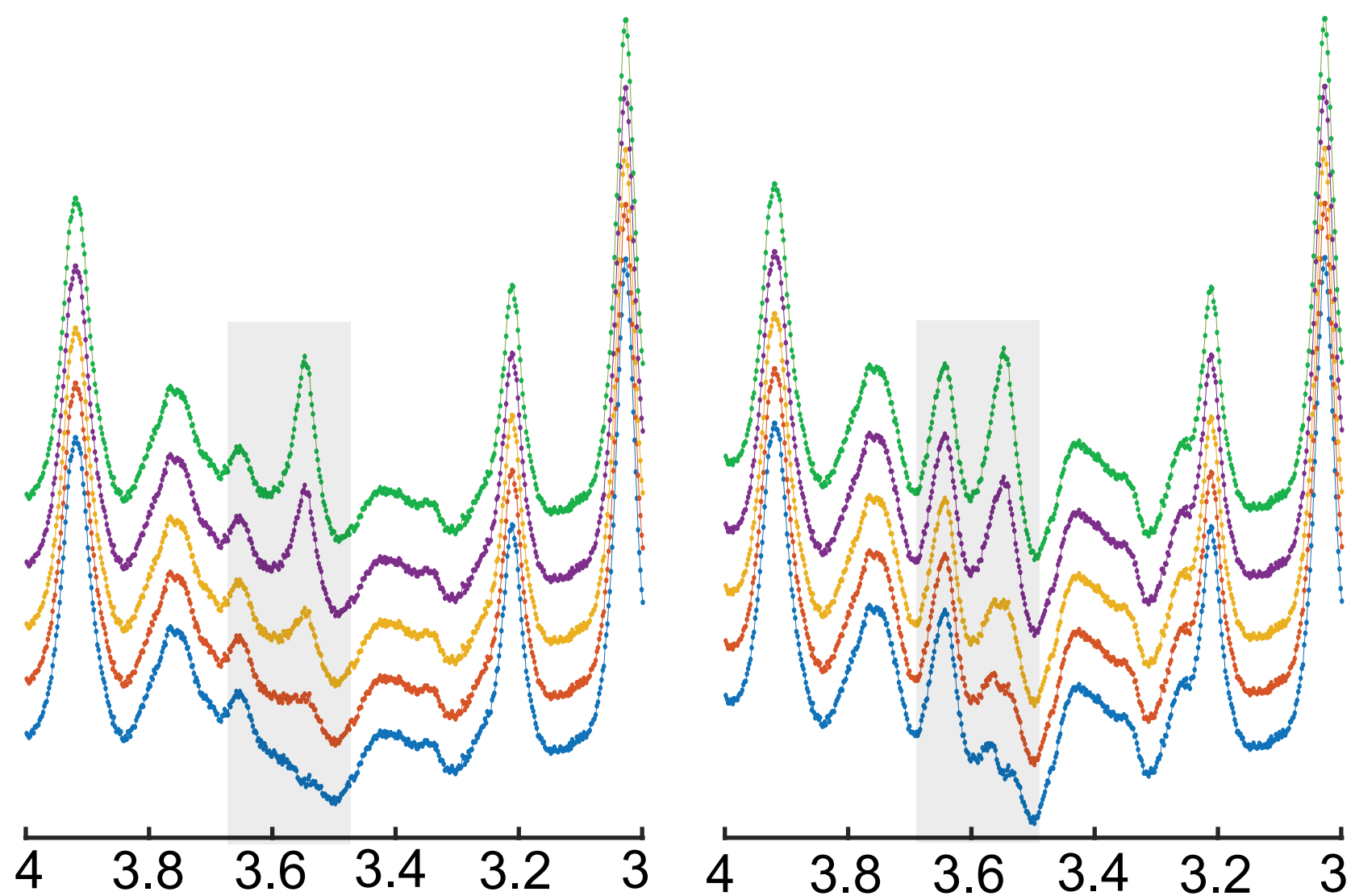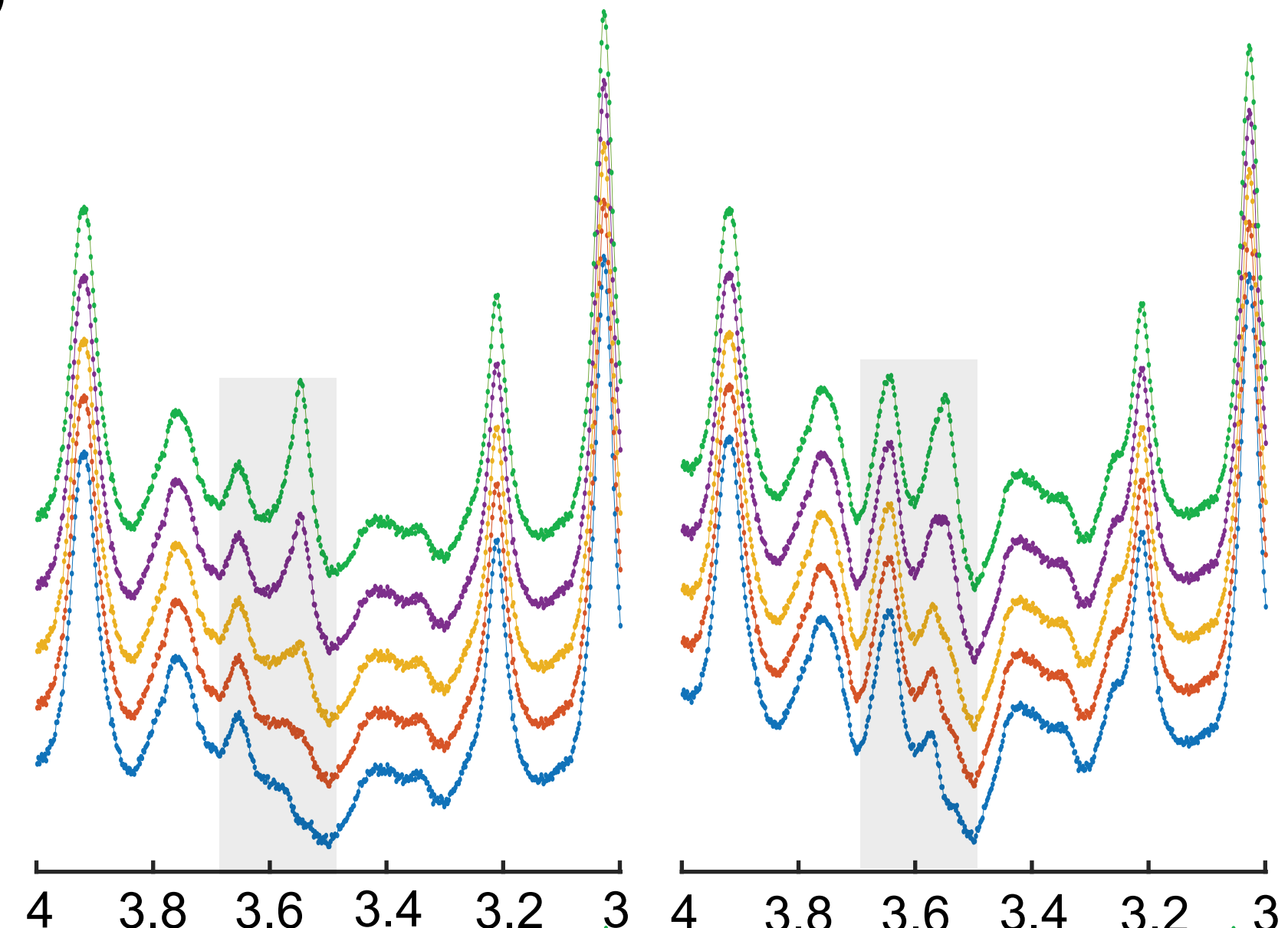

10 Hz

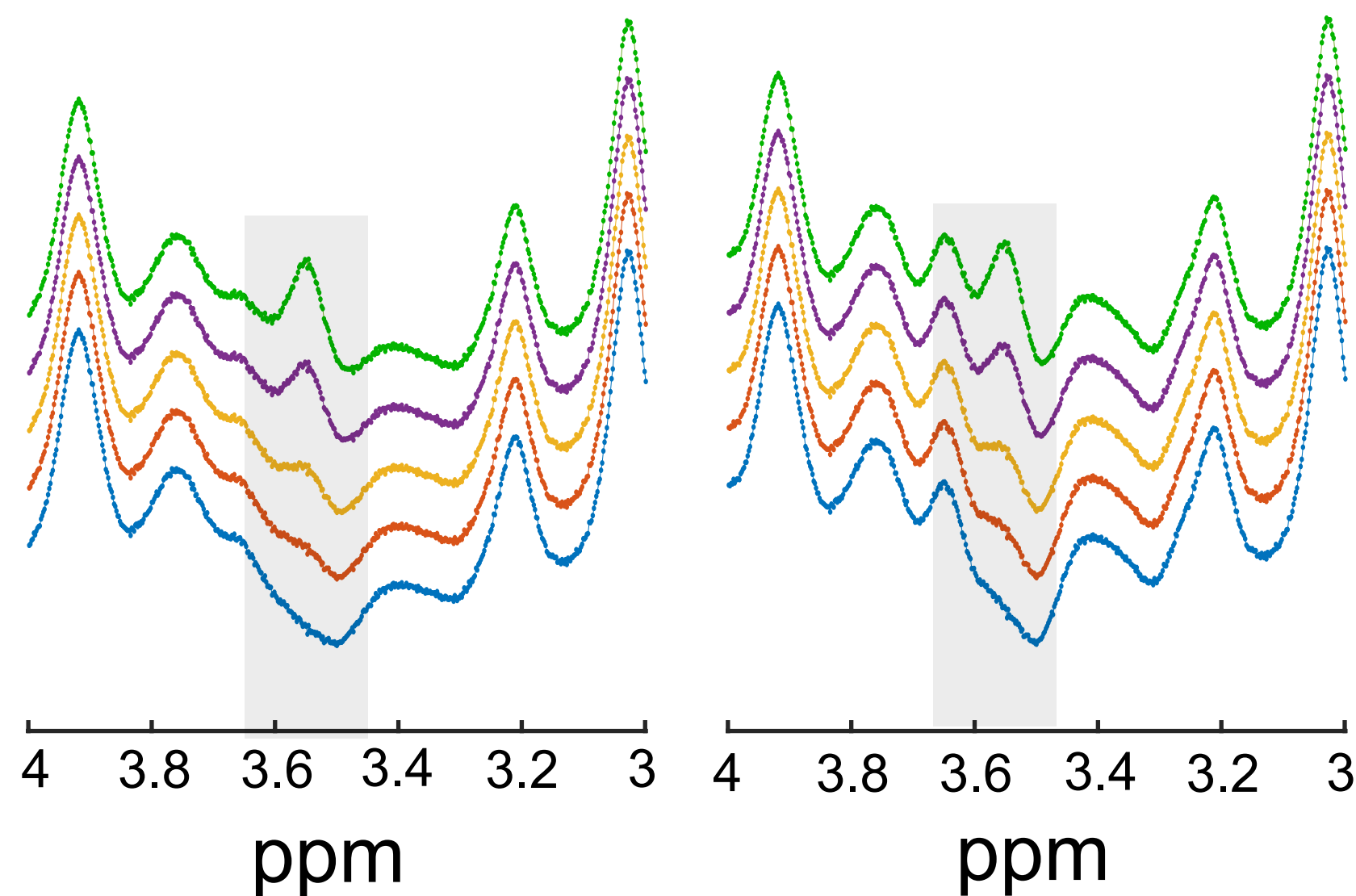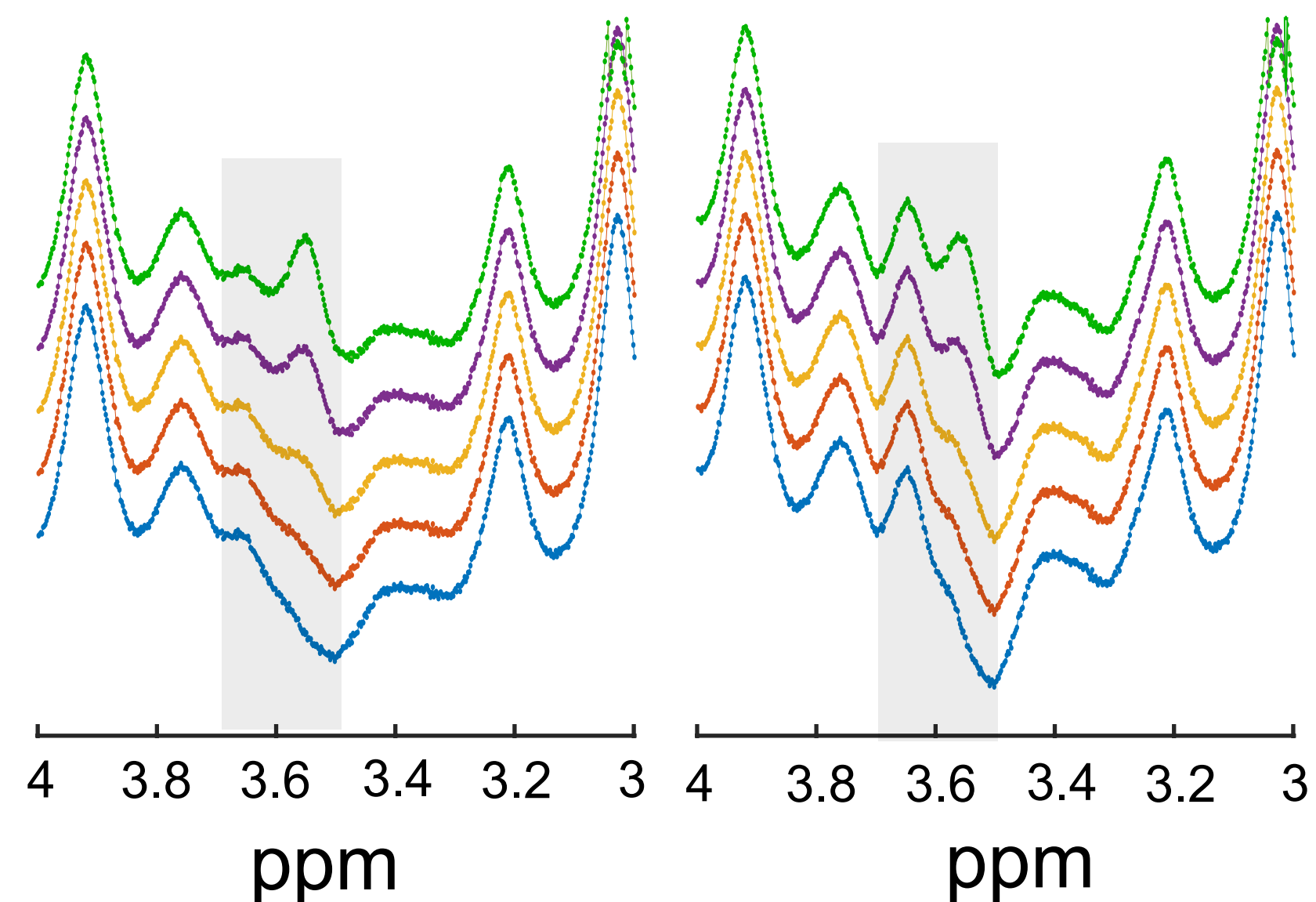

Supplement: Supplementary file 1 — Table S1: Minimum Reporting Standards in Magnetic Resonance Spectroscopy checklist. Figure S1: Simulated MEGA‐PRESS SUM spectra as a function of TE, line broadening, and Gly/mI concentration ratios for (A) TE 64 ms and (B) TE 68 ms. All metabolites were scaled to in vivo concentrations except for Gly which were scaled to values of 0, 0.5, 1, 2, or 3 mM, and mI, which were scaled to values of 3 (left column) or 10 mM (right column). Each simulation was line‐broadened to either 5 Hz (top row) or a 10 Hz (bottom row). All spectra are shown from 3 to 4 ppm. The highlighted regions are to mark notable differences in Gly and mI signal. [file MRM-95-2479-s001.zip › mrm70219-sup-0002-FigureS1.pdf]
